# Surgery

**Published:** 1905-09

**Authors:** T. Carwardine


### SURGERY.

During the last ten years **prostatectomy** has become an established operation, and with a more accurate appreciation of the pathology brought about by surgical aggressiveness there has been a diminution of mortality following the operation, and an increase of its scope of utility, particularly in the hands of certain surgeons who seem endowed with natural aptitude for success.

A recent number of the *Annals of Surgery*[2] was devoted almost entirely to this subject, and we may profitably analyse the views of our American brethren who have kept to the front in this work, more particularly because they have a leaning towards the perineal route of operation, like French surgeons; the tendency of British surgeons being towards the suprapubic route from the able advocacy of McGill and Freyer.

*Some Points in Pathology.*—Dr. P. M. Pilcher has made a study of twenty-three prostates removed by operation, and refers to the fact that observers, or cases, differ as to the amount of glandular elements present from one-third to five-sixths of the substance of the gland. He finds that prostates removed for urinary obstruction present three types, each of which may be present whether a history of gonorrhœa or cystitis be obtainable or not. In the greatly *enlarged soft prostate*, the illustrations of which are now quite familiar to all medical readers, the anatomical proportions are very variable; but the mucous membrane lining the prostatic urethra is intimately related to the gland itself, and can only be separated from it with difficulty. The *atrophic* form, on the other hand, may be even smaller than normal, yet retention is a prominent clinical feature in such cases. The third variety is of a *mixed* type, intermediate between the other two. Microscopically, the most striking change from the normal is the relative and absolute increase in the amount of glandular tissue in parts with cyst-formation; and the ducts are filled with retained secretion, and often with degenerated epithelium, leucocytes, amylaceous bodies, and calculi. With this there is increase of the connective tissue and muscular structures to a variable extent, with small round-celled infiltration, and sometimes areas of extravasated blood.

We shall refer to the question of potency shortly, but from an anatomical standpoint F. S. Watson states that the level of the lower border of the urethral aspect of each lateral lobe is a little above that of the floor of the prostatic urethra, and consequently above the level of the ejaculatory ducts. Any operation, therefore, which does not involve the floor of the prostatic urethra will not injure the ducts. In the case of a middle lobe being present, it is intravesical, and outside the sphere occupied by the ducts.

*Contraction of the Neck of the Bladder as a Cause of Prostatism without Enlargement of the Prostate.*—Dr. Charles H. Chetwood describes a condition which is perhaps not fully recognised, although we do not think the examples in his tables of thirty-six cases should all come under that head. He says that contracture of the neck of the bladder is a common cause of the train of symptoms of prostatism, characterised by urgency and frequency of urination, with pain and more or less retention. The contracture is simply a fibrous stenosis of the vesical orifice, induced by chronic inflammation, and often gonorrhœal in origin. The observation is not new, for Sir Henry Thompson recognised in 1883 that there could be obstruction to the bladder outlet without prostatic hypertrophy. The condition has also been recently noticed by French observers, and must be borne in mind as a common cause for vesical obstruction, and a frequent explanation for "prostatism without enlargement of the prostate."

*Has the Catheter a Place in Treatment?*—This question has to be discussed in all seriousness in the present day. P. Thorndike writes that it is his custom to have long talks with these patients, and to offer a choice of the two methods of procedure, giving his bias in favour of the catheter for the immediate future. He protests against the catheter being carelessly thrown aside for procedures of a more radical kind, although the day may come for the performance of prophylactic prostatectomy. F. S. Watson states, on the other hand, "the really essential point for those who are experts in this special province is to bring home the fact to the profession at large that the operative treatment of prostatic hypertrophy has been brought to a sufficient degree of perfection to make it evident that the patients should be given the benefit of it, and should not be submitted to the dangers of catheterism as they have been in the past." He believes that the mortality attending the catheter treatment is even greater, except in the class of people of the best social status, than that attending prostatectomy, and that the mortality of perineal prostatectomy, were it practised at a sufficiently early age of the malady, would certainly prove to be far less than that associated with the catheter treatment in all classes of persons. L. S. Pilcher says, "Shall not the use of the catheter first be tried?" Certainly as a temporary resort, but, in the light of present experience, always rather under protest than as a measure possessing the full recommendation of the surgeon. There is probably a judicious mean, and if the results of operation were universally as successful as they are in the published series of a few operators no doubt the profession at large would soon toe the line. But the standard in surgery will always be the consistent average results, and not the ideal best results of a few experts. Manual dexterity counts for much in prostatectomy, and no surgeon seems to have published cases in which he has failed to remove the prostate; and half-measures are, as a rule, worse than useless.

*The Question of Priority.*—The claimants to surgical priority are very jealous of their claims, and are apt to vie with little Jack Horner in their egotistical conclusions. The prostate has proved a surgical plum, and several have been the claimants to priority in pulling it out with fabulous self-satisfaction. Dr. Francis S. Watson gives Gouley, of New York, the credit for originally and definitely proposing perineal prostatectomy in a publication in 1873: "Gouley's operation is that of the rapid finger enucleation done through the sides of the prostatic urethra by the forefinger tip introduced into the latter through an ordinary external perineal urethrotomy incision. It is the operation which I have practised from time to time since 1889, having learned it from the verbal instructions of Dr. Gouley in 1884. It is the method which I still prefer."

With regard to suprapubic prostatectomy, it does not now appear that Mr. Freyer's claim to an original operation is justified by the facts. Watson figures a complete prostate, which he removed in one mass, together with the prostatic urethra by the suprapubic route in 1897, or some three or four years before Freyer published his operation. "The fact that I removed the hypertrophied gland in one mass, together with the prostatic urethra, did not strike me then, nor does it seem to me now, to constitute a new procedure that could be considered as distinctive in any essential sense of the word from the original suprapubic prostatectomy of McGill." "The credit of suprapubic prostatectomy belongs to Belfield and McGill." Eugene Fuller lays claim to having performed the operation, and gives photographs of specimens prior to Freyer. He published his paper in New York, in June, 1895. His discussion is too long to follow in detail, but his assertions are interesting and of such a character that Freyer must either withdraw his claim or meet the statements. Briefly they are as follows: In the spring of 1900 Fuller published a book incorporating his paper of 1895. In August, 1900, Dr. R. Guiteras read a paper in Paris before the International Medical Congress giving his slight modification of Fuller's operation, that of introducing one or two fingers into the rectum. On his way to Paris he called on Mr. Freyer and fully acquainted him with Fuller's operation and his own modification. Guiteras wrote: "Dr. Freyer was very much pleased with the description of the operations, and said that he would try the method." Freyer's first case of suprapubic prostatectomy entered hospital shortly after Dr. Guiteras's instructive visit, and Freyer, showing himself an apt student, operated successfully, following the exact method taught him by the New York surgeon. This is the gist of the argument, and for further facts in its support the paper may be consulted.

*Anæsthesia.*—According to Pilcher chloroform is in general to be preferred for the aged. Young has reported ten cases with the use of spinal cocainisation without unpleasant sequelæ, and Tinker has removed two prostates under local anæsthesia with eucaine and adrenalin. Lilienthal says in most cases nitrous oxide may be used at first, and it will frequently suffice, but may be followed by ether if necessary. J. Wiener, jun., has operated on ten cases under nitrous oxide alone without mortality, the average time taken being ten minutes, and the extremes five and nineteen minutes. He grants that a good deal of physical strength and some experience are needed.

*Preliminary Cystoscopy.*—There is common agreement that this is unnecessary if the suprapubic operation be performed, but the majority of those who prefer the perineal route think cystoscopy advisable. Lilienthal and Wiener, who prefer the suprapubic operation, deprecate cystoscopy. Pilcher, who describes a perineal operation on the lines of the technique of Proust, disapproves cystoscopy. On the other hand, in connection with the perineal operation, Young says: "I wish to state again my belief in the great advisability of a careful preliminary cystoscopic examination. In this way only have I been saved from several serious blunders." A very complete description of the cystoscopes and their uses is given by J. Cunningham, jun.

*The choice of route—suprapubic or perineal?*—On the whole, the opinion seems to be equally divided as to the preferable route. Young and Pilcher prefer the perineal method, Lilienthal and Ruggles prefer the suprapubic, whilst Watson and Fuller perform both operations, the former doing the perineal operation in two-thirds of his cases, and the latter the suprapubic operation in the same proportion of the whole.

*Mortality.*—This is probably represented in its most favourable aspects. Eugene Fuller has had a mortality of late of only 4 per cent. for the better class of patients, and $5\frac{1}{2}$ to $6\frac{1}{2}$ per cent. for hospital patients. Young has had four deaths out of 75 operations, and Pilcher two out of 23. A mortality of 5 to 10 per cent. must be regarded as the minimum, the perineal operation probably having the smaller mortality. Pilcher has collected the statistics of several operators, and they work out thus :—Small median perineal incision with finger enucleation unaided by sight — 162 cases, five deaths ; free transverse perineal incision, enucleation under visual control—220 cases, ten deaths; suprapubic enucleation (Freyer)—107 cases, five deaths.

*Prostatectomy and Potency.*—There is general agreement that if care be taken so as to avoid injury to the floor of the urethra the potency of the individual is not lessened as a rule, and in some cases it is improved when previously impaired. If, however, the operation be not carefully performed, then potency ceases. To preserve the sexual powers as far as possible it would seem desirable in the suprapubic operation to enucleate the separate lobes from within the commissural capsule, rather than the prostate as a whole with its attached urethra; and in the perineal operation to avoid injury to the floor of the prostatic urethra by keeping the finger with its back towards the urethra and enucleating upwards and outwards. Horwitz has advanced the theory that impotency may follow prostatectomy independently of injury to the ducts, due to disturbance of the important nervous mechanism of the gland by the trauma.

*Restoration to Voluntary Micturition.*—Young, in a series of seventy-five cases, states that they can all void urine naturally, and that in only two cases is there any residual urine. Pilcher states that in over 60 per cent. of cases permanent restoration of the function results : he had one case of only partial relief out of 23.

*Continence.*—Ruggles concludes that incontinence is due to injury to the sphincter urethræ muscle at the level of the membranous urethra. It is therefore only likely to be met with after the perineal operation. Young has found in seventy-five cases that incontinence is only temporary, not permanent. In one case incontinence persisted in the day-time. Pilcher had defective continence in two cases out of 23. Ruggles estimates the permanent incontinence as about 5 per cent., and states that the percentage has been rather high at Rochester (7½ per cent.).

*Stricture.*—This was formerly feared, but facts do not support the theory. Young has not personally observed any case, and in spite of the inevitable injury to the urethra Pilcher does not regard it as a sequel to be reckoned upon.

*Fistulæ.*—These may be suprapubic, perineal, or recto-urethral, and here the disadvantage seems to be with the perineal operation. Suprapubic fistulæ do not seem to give trouble, and heal sooner or later if they occur. Perineal fistulæ persisted in two out of Young's 75 cases. More serious, however, is the recto-urethral fistula, due to excessive perineal traumatism or sloughing from gauze drainage. Young had four cases, and was only able to cure them by first performing suprapubic cystotomy for temporary bladder drainage. It occurred to Pilcher on three occasions, and in one case it could not be cured, two-thirds of the urine passing into the rectum with each micturition, and being subsequently voided at stool.

*Epididymitis and Orchitis.*—These may follow either the suprapubic or perineal operations. In Pilcher's 23 cases it occurred six times, and ended in suppuration in two.

On the whole, therefore, it would seem that although the perineal operation has a lower mortality, it is subject to greater disabilities, and the functional results are not so constantly perfect as with the suprapubic operation. It is therefore probable that both operations will continue to be performed in the future, until it may be possible to diminish the disabilities following the perineal procedure.

T. CARWARDINE.

